# Supplementary material for: Characterization of trade-offs between immunity and reproduction in the coral species Astrangia poculata
Source: PeerJ. 2023 Dec 4;11:e16586. doi: 10.7717/peerj.16586 (PMC10702360; doi:10.7717/peerj.16586)
Supplement: Supplemental Information 1 [file peerj-11-16586-s001.docx]

**Cell-Free Extract Generation**

Tissues for protein extraction were removed from the remaining portion of the *A. poculata* colony via airbrushing with 100 mM Tris + 0.5 mM DTT (pH 7.8). The resultant slurry was homogenized for one minute, followed by a 10 minute incubation on ice. One milliliter of resulting slurry was reserved for melanin concentration estimation, stored at -20°C. The remaining volume was centrifuged for 5 minutes (2250 RCF at 4°C). The resultant supernatant was stored at -80°C for later analyses.

Immune, lipid, and carbohydrate assays were performed using preserved host-enriched protein and melanin aliquots from the cell-free extract. Colorimetric immune assays were run in triplicate on 96 well plates using a Cytation 1 cell imaging multi-mode reader and Gen 5 software (BioTek Instruments, Winooski, VT, USA). A Red660 (G Biosciences, St. Louis, Missouri) assay was used to determine protein concentration to standardize immune activity metrics (Mydlarz & Palmer 2011).

**Prophenoloxidase cascade assays (Total PO and Melanin)**

Total phenoloxidase activity (prophenoloxidase + phenoloxidase) was measured by diluting 20µl of coral protein extract in 20µl of 50 mM PBS (pH 7.0) in a 96 well plate (Costar, Corning, Kennebunk, ME). In order to convert prophenoloxidase to phenoloxidase, wells were then incubated with 25µl of trypsin (0.1 mg/mL DI) for 30 minutes at room temperature. Negative controls were run in duplicate using extraction buffer (100 mM Tris + 0.5 mM DTT [pH 7.8]) in place of sample. Following incubation, 30µl of dopamine (10 mM) was added to each sample to initiate the reaction. Immediately after addition of dopamine, absorbance was read every minute for 20 minutes at 490 nm. Total PO activity was calculated as change of absorbance (final – initial) per milligrams protein per minute, at the steepest point of the curve, standardized by protein concentration (Mydlarz & Palmer 2011).

Tissue samples for melanin analysis were desiccated in a SpeedVac (Eppendorf, Vacufuge plus). To calculate melanin concentration, a spatula full of 10 mm glass beads (~200µl) was added to each tube, and vortexed for 10 seconds. Next, 400µl of 10M NaOH were added, and samples were vortexed again for 20 seconds. Samples then underwent a 48 hour incubation period, with vortexing at 24 and 48 hours. At the end of this period, tubes were centrifuged at 183 RCF for 10 minutes at room temperature. Forty microliters of the supernatant were transferred to a ½ volume 96-well UV plate (UV-STAR, Greiner bio-one, Frickenhausen, Germany). A standard curve of melanin dissolved in 10 M NaOH and processed identically to the samples was used to convert absorbance readings to melanin concentration (Mydlarz & Palmer 2011). Absorbance was recorded at 410 and 490 nm. Melanin concentrations were calculated using a melanin standard (Sigma Aldrich, St. Louis, Missouri) and standardized to total dry tissue weight (mg melanin/mg tissue).

**Antioxidant assays**

Following previously established protocols, activity was assessed for two antioxidants: catalase (CAT) and peroxidase (POX; (Fuess et al. 2016; Mydlarz & Palmer 2011)). Catalase activity was measured by adding 5µl of sample protein extract to a 96-well UV well plate (UV-STAR, Greiner bio-one, Frickenhausen, Germany), which was diluted with 45µl of 50 mM PBS (pH 7.0). Seventy-five microliters of 25 mM H_2_O_2_ were added to each well to initiate the reaction. Absorbance was immediately measured at 240 nm every 30 seconds for 15 minutes. A standard curve of hydrogen peroxide was used to calculate H_2_O_2_ concentrations. Catalase activity was calculated as H_2_O_2_ scavenged per min at the steepest point of the curve, normalized to protein concentration (Fuess et al. 2016; Mydlarz & Palmer 2011).

Peroxidase activity was measured by diluting 20µl of sample protein extract with 20µl of 10 mM PBS (pH 6.0) in a 96-well plate (Costar, Corning, Kennebunk, ME). Then, 25µl of 5 mM guaiacol in 10 mM PBS (pH 6.0) was added. Negative controls were run in duplicate using extraction buffer (100 mM Tris + 0.5 mM DTT (pH 7.8)) in place of protein extract. To initiate the reaction, 20µl of 20 mM H_2_O_2_ was pipetted into each well. Absorbance was read at 470 nm every minute for 15 minutes. Activity was calculated as change in absorbance per minute at the steepest point of the curve, normalized to protein concentration (Mydlarz & Harvell 2007).

**Antibacterial activity**

Antibacterial activity was assessed against *Vibrio* *coralliilyticus* (Strain RE22Sm provided by D. Nelson at University of Rhode Island) grown in Luria Broth. *V. coralliilyticus* is a known cnidarian pathogen with roles in numerous coral disease (Ushijima et al. 2020; Ushijima et al. 2014). In a sterile hood, 140µl of *V. coralliilyticus* diluted to a optical density of 0.2 at 600 nm and 60µl of sample diluted to a standard protein concentration were added to wells of a sterile 96-well plate. The plate was incubated for 6 hours at 27°C, with absorbance at 600 nm recorded every 10 minutes. Bacterial growth rates were determined as the change in absorbance during the logarithmic growth phase (Pinzon et al. 2014).

**Total Lipid Content**

A standard protocol for quantification of lipids within coral tissue slurries was used to estimate lipid content (Bove & Baumann 2021). Aliquots of 150µl of sample extract were desiccated overnight in a SpeedVac (Eppendorf, Vacufuge plus). Five hundred microliters of a 2:1 chloroform/methanol mixture and 100µl of 0.05 M NaCl were added to each desiccated sample. Samples were vortexed to dissolve lipids, and were placed on a shaker (Corning LSE Benchtop Shaking Incubator) for one hour, vortexing every 15 minutes. Samples were then centrifuged at 1650 RCF for 5 minutes. One hundred microliters of the bottom layer of the centrifuged samples were then added to a 96-well PCR plate in triplicate. Fifty microliters of methanol were added to all wells and placed in a hot water bath (70°C) for 15 minutes. Following solvent evaporation, 100µl of 18 M sulfuric acid were added to each well, and placed in a thermocycler (Eppendorf Mastercycler Nexus) at 90°C for 20 minutes followed by 4°C for 20 minutes. Seventy-five microliters of sample were transferred from each PCR well into a new 96 well plate. Following an initial absorbance reading at 540 nm, 34.5µl of 0.2 mg/mL vanillin in 17% phosphoric acid were added to each well, and incubated in the dark at room temperature for 5 minutes, after which absorbance was read again (Cheng et al. 2011). The difference between the resulting absorbance was converted to lipid concentration (mg/mL) using a standard curve of corn oil diluted in chloroform treated identically to samples. Final values were standardized based on dry tissue weight (lipids/ug tissue).

**Total Carbohydrate Content**

Total carbohydrate concentration was estimated following previously established protocols (Dubois et al. 1956; Masuko et al. 2005). Fifty microliters of sample extract, blank, or glucose standard were added in triplicate to a 96 well plate. 150µl of H_2_SO_4_ were added to each well, immediately followed by 30µl of 5% phenol. The plate was incubated in an uncovered hot water bath (70°C) for 5 minutes, and cooled for 15 minutes. Absorbance was read at 485 nm and 750 nm, and then the absorbance values at 750 nm were subtracted from the absorbance values at 485 nm to correct for differential scattering (Fu et al. 2008). A serial dilution of glucose, treated identically to samples, was used to convert absorbance to total carbohydrate concentration.

**REFERENCES CITED**

Bove CB, and Baumann J. 2021. Coral Lipid Assay for 96-well plates. *protocolsio*. 10.17504/protocols.io.bvcfn2tn

Cheng YS, Zheng Y, and VanderGheynst JS. 2011. Rapid quantitative analysis of lipids using a colorimetric method in a microplate format. *Lipids* 46:95-103. 10.1007/s11745-010-3494-0

Dubois M, Gilles KA, Hamilton JK, Rebers Pt, and Smith F. 1956. Colorimetric method for determination of sugars and related substances. *Analytical chemistry* 28:350-356.

Fu F-X, Zhang Y, Warner ME, Feng Y, Sun J, and Hutchins DA. 2008. A comparison of future increased CO2 and temperature effects on sympatric Heterosigma akashiwo and Prorocentrum minimum. *Harmful Algae* 7:76-90. 10.1016/j.hal.2007.05.006

Fuess LE, Pinzomicronn CJ, Weil E, and Mydlarz LD. 2016. Associations between transcriptional changes and protein phenotypes provide insights into immune regulation in corals. *Dev Comp Immunol* 62:17-28. 10.1016/j.dci.2016.04.017

Masuko T, Minami A, Iwasaki N, Majima T, Nishimura S, and Lee YC. 2005. Carbohydrate analysis by a phenol-sulfuric acid method in microplate format. *Anal Biochem* 339:69-72. 10.1016/j.ab.2004.12.001

Mydlarz LD, and Harvell CD. 2007. Peroxidase activity and inducibility in the sea fan coral exposed to a fungal pathogen. *Comp Biochem Physiol A Mol Integr Physiol* 146:54-62. 10.1016/j.cbpa.2006.09.005

Mydlarz LD, and Palmer CV. 2011. The presence of multiple phenoloxidases in Caribbean reef-building corals. *Comp Biochem Physiol A Mol Integr Physiol* 159:372-378. 10.1016/j.cbpa.2011.03.029

Pinzon CJ, Dornberger L, Beach-Letendre J, Weil E, and Mydlarz LD. 2014. The link between immunity and life history traits in scleractinian corals. *PeerJ* 2:e628. 10.7717/peerj.628

Ushijima B, Meyer JL, Thompson S, Pitts K, Marusich MF, Tittl J, Weatherup E, Reu J, Wetzell R, Aeby GS, Hase CC, and Paul VJ. 2020. Disease Diagnostics and Potential Coinfections by Vibrio coralliilyticus During an Ongoing Coral Disease Outbreak in Florida. *Front Microbiol* 11:569354. 10.3389/fmicb.2020.569354

Ushijima B, Videau P, Burger AH, Shore-Maggio A, Runyon CM, Sudek M, Aeby GS, and Callahan SM. 2014. Vibrio coralliilyticus strain OCN008 is an etiological agent of acute Montipora white syndrome. *Appl Environ Microbiol* 80:2102-2109. 10.1128/AEM.03463-13
