# Supplementary material for: Characterization of trade-offs between immunity and reproduction in the coral species Astrangia poculata
Source: PeerJ. 2023 Dec 4;11:e16586. doi: 10.7717/peerj.16586 (PMC10702360; doi:10.7717/peerj.16586)
Supplement: Supplemental Information 4 — Best-fit linear models for sperm density when including symbiotic state, carbohydrate concentration, lipid concentration as predictors. All possible models were compared and model averaging was used where appropriate (AIC delta < 2). Asterisks (*) represent significant p-value (a = 0.05). [file peerj-11-16586-s004.docx]

| **Predictors** | **Estimates** | **SE** | **SEadj** | **z** | **p value** |
| --- | --- | --- | --- | --- | --- |
| (Intercept) | 12.2 | 0.687 | 0.789 | 15.4 | **< 0.001 ***** |
| Carbohydrates | 218.9 | 85.1 | 91.7 | 2.39 | **0.0169*** |
| Symbiotic State | 0.229 | 0.515 | 0.538 | 0.427 | 0.670 |
