# Supplementary material for: Characterization of trade-offs between immunity and reproduction in the coral species Astrangia poculata
Source: PeerJ. 2023 Dec 4;11:e16586. doi: 10.7717/peerj.16586 (PMC10702360; doi:10.7717/peerj.16586)
Supplement: Supplemental Information 5 — Best-fit linear models for each immune parameter when including symbiotic state, carbohydrate concentration, lipid concentration, and sperm produced per polyp as predictors. All possible models were compared and model averaging was used where appropriate (AIC delta < 2). Asterisks (*) represent significant p-value (a = 0.05). [file peerj-11-16586-s005.docx]

| **Catalase** | | | | | |
| --- | --- | --- | --- | --- | --- |
| **Predictors** | **Estimates** | **SE** | **SEadj** | **z** | **p value** |
| (Intercept) | 1,386 | 134.5 | 141.5 | 9.80 | **< 0.001 ***** |
| Sperm | -1.10E^-5^ | 2.02E^-5^ | 2.10E^-5^ | 0.529 | 0.597 |
| Carbohydrates | 9,829 | 18,200 | 18,820 | 0.522 | 0.602 |
| **Peroxidase** | | | | | |
| **Predictors** | **Estimates** | **SE** | **SEadj** | **z** | **p value** |
| (Intercept) | 1.25 | 0.479 | 0.501 | 2.50 | **0.0123** |
| Symbiotic State | 0.658 | 0.622 | 0.644 | 1.02 | 0.307 |
| **Antibacterial Activity** | | | | | |
| **Predictors** | **Estimates** | **SE** | **SEadj** | **z** | **p value** |
| (Intercept) | 3.35 | 0.187 | 0.196 | 17.2 | **< 0.001 ***** |
| Carbohydrates | 13.5 | 20.3 | 20.9 | 0.647 | 0.518 |
| Lipids | 5.32 | 15.0 | 15.5 | 0.343 | 0.732 |
| **Melanin** | | | | | |
| **Predictors** | **Estimates** | **SE** | **SEadj** | **z** | **p value** |
| (Intercept) | 5.01^-5^ | 1.92E^-5^ | 2.06E^-5^ | 2.44 | **< 0.001 ***** |
| Carbohydrates | 5.08E^-3^ | 2.11E^-3^ | 2.29E^-3^ | 2.22 | **0.026*** |
| Lipids | -1.24E^-5^ | 1.77E^-5^ | 1.83E^-5^ | 0.675 | 0.500 |
