# Supplementary figures and images for: Characterization of trade-offs between immunity and reproduction in the coral species Astrangia poculata
Source: PeerJ. 2023 Dec 4;11:e16586. doi: 10.7717/peerj.16586 (PMC10702360; doi:10.7717/peerj.16586)

**Symbiont Density**  
(cells/area)

4e<sup>5</sup>  
3e<sup>5</sup>  
2e<sup>5</sup>  
1e<sup>5</sup>  
0

White

Brown

**Symbiotic State**

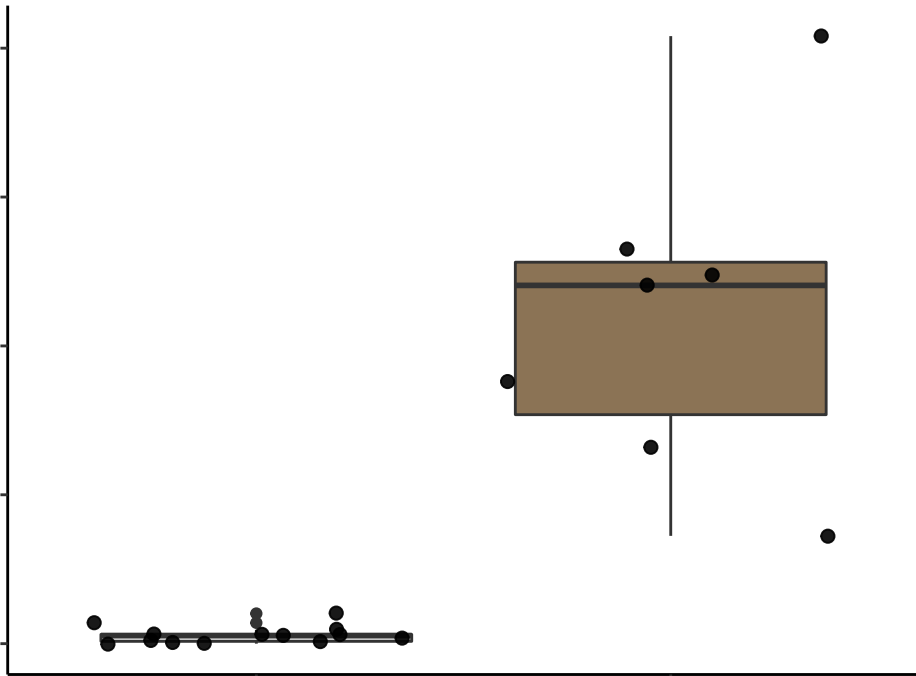

Supplement: Supplemental Information 6 — Dots represent raw data values within each group. Colonies classified in the brown symbiotic state have significantly higher symbiont density than those classified as white (Wilcoxin Test, p < 0.001) [file peerj-11-16586-s006.pdf]
